# Supplementary material for: Multi‐dataset identification of innovative feature genes and molecular mechanisms in keratoconus
Source: J Cell Mol Med. 2024 Sep 19;28(18):e70079. doi: 10.1111/jcmm.70079 (PMC11412914; doi:10.1111/jcmm.70079)
Supplement: Supplementary file 2 — Material S2. [file JCMM-28-e70079-s002.zip › 7.GSEA analysis/3.DBNDD1/KEGG_NITROGEN_METABOLISM.html]

Details for gene set KEGG\_NITROGEN\_METABOLISM[GSEA]

|  || Dataset | DBNDD1.group.cls#h\_versus\_l |
| Phenotype | group.cls#h\_versus\_l |
| Upregulated in class | l |
| GeneSet | KEGG\_NITROGEN\_METABOLISM |
| Enrichment Score (ES) | -0.5760633 |
| Normalized Enrichment Score (NES) | -1.4867672 |
| Nominal p-value | 0.064453125 |
| FDR q-value | 0.7079825 |
| FWER p-Value | 0.81 |
Table: GSEA Results Summary

  

Fig 1: Enrichment plot: KEGG\_NITROGEN\_METABOLISM      
 Profile of the Running ES Score & Positions of GeneSet Members on the Rank Ordered List

  

| SYMBOL | TITLE | RANK IN GENE LIST | RANK METRIC SCORE | RUNNING ES | CORE ENRICHMENT || 1 | CA5A | na | 5279 | 0.171 | -0.0937 | No |
| 2 | CA3 | na | 7558 | 0.112 | -0.1209 | No |
| 3 | CA6 | na | 8203 | 0.100 | -0.1054 | No |
| 4 | GLS2 | na | 12449 | 0.043 | -0.2130 | No |
| 5 | CA7 | na | 14180 | 0.029 | -0.2531 | No |
| 6 | GLUD1 | na | 15404 | 0.020 | -0.2815 | No |
| 7 | GLUL | na | 17464 | 0.006 | -0.3386 | No |
| 8 | CA13 | na | 19027 | -0.005 | -0.3820 | No |
| 9 | CPS1 | na | 19099 | -0.005 | -0.3823 | No |
| 10 | CA9 | na | 19736 | -0.011 | -0.3970 | No |
| 11 | CA8 | na | 20542 | -0.018 | -0.4142 | No |
| 12 | GLS | na | 21167 | -0.023 | -0.4241 | No |
| 13 | GLUD2 | na | 23554 | -0.050 | -0.4757 | No |
| 14 | CA14 | na | 27039 | -0.109 | -0.5389 | Yes |
| 15 | CA5B | na | 27296 | -0.114 | -0.5072 | Yes |
| 16 | CA4 | na | 28858 | -0.153 | -0.5000 | Yes |
| 17 | ASNS | na | 29590 | -0.175 | -0.4612 | Yes |
| 18 | CA12 | na | 29923 | -0.186 | -0.4071 | Yes |
| 19 | HAL | na | 30637 | -0.209 | -0.3563 | Yes |
| 20 | CTH | na | 31662 | -0.247 | -0.3015 | Yes |
| 21 | CA1 | na | 33149 | -0.341 | -0.2280 | Yes |
| 22 | AMT | na | 33334 | -0.356 | -0.1117 | Yes |
| 23 | CA2 | na | 34111 | -0.445 | 0.0178 | Yes |
Table: GSEA details [plain text format]

  

Fig 2: KEGG\_NITROGEN\_METABOLISM      
 Blue-Pink O' Gram in the Space of the Analyzed GeneSet

  

Fig 3: KEGG\_NITROGEN\_METABOLISM: Random ES distribution      
 Gene set null distribution of ES for **KEGG\_NITROGEN\_METABOLISM**
